# The modified Atkins diet in children with Prader-Willi syndrome

**DOI:** 10.1186/s13023-020-01412-w

**Published:** 2020-06-03

**Authors:** Grace Felix, Eric Kossoff, Bobbie Barron, Caitlin Krekel, Elizabeth Getzoff Testa, Ann Scheimann

**Affiliations:** 1 Division of Pediatric Gastroenterology and Nutrition, Johns Hopkins School of Medicine, Baltimore, MD USA; 2 Pediatric Specialists of Virginia/INOVA Children’s Hospital, Fairfax, Virginia USA; 3 Department of Pediatric Neurology, Johns Hopkins School of Medicine, Baltimore, MD USA; 4 Institute of Clinical and Translational Research, Johns Hopkins School of Medicine, Baltimore, MD USA; 5 Department of Psychology, Center for Pediatric Weight Management & Healthy Living, Mt. Washington Pediatric Hospital, Baltimore, MD USA; 6 Division of Pediatric Gastroenterology and Nutrition, Johns Hopkins Hospital, 600 N. Wolfe Street Brady 320, Baltimore, MD 21287–2631 USA

**Keywords:** Prader-Willi syndrome, Pediatric obesity, Obesity, Diet, Low-carbohydrate diet, Ketogenic

## Abstract

**Background:**

Prader-Willi Syndrome (PWS) is the most common genetic cause of obesity. Various dietary strategies have been used for weight management for people with PWS.

**Methods:**

This was a clinical feasibility study to test the use of the Modified Atkins Diet (low carbohydrate and high fat) for children with PWS ages 6–12 years who were overweight/obese. Participants went on the Modified Atkins Diet for 4 months and then returned to have anthropometry repeated including repeat labs and behavior questionnaires.

**Results:**

Seven children (ages 6–12) were enrolled in the study. Four participants completed the 4-month diet trial; two were unable to comply with the diet and stopped prematurely. One patient lost 2.9 kg; the others maintained their weight. Adverse effects were increases in LDL (expected based on larger studies) and hypercalciuria (with no renal stones) for one patient. Positive effects on hyperphagia and behavior were noted subjectively by families.

**Conclusion:**

The Modified Atkins Diet can be a feasible low carbohydrate option for children with Prader-Willi Syndrome for weight management. Long-term use of the diet in patients with Prader-Willi Syndrome needs to be studied further.

## Introduction

Prader-Willi Syndrome (PWS) is the most common genetic cause of obesity caused by mutations in the paternal genes on chromosome 15q11-q13. In the 20 years after this syndrome was first described by Drs. Prader and Willi in 1956, its incidence was estimated to be 1 in 25,000 births. However, with earlier and increased diagnosis, it is now thought that the incidence is closer to 1 in 15,000 [1, 2]. Classically, it presents in infancy with the combination of hypotonia, feeding problems, failure to thrive, developmental delay, and hypogonadism/hypogenitalism [3, 4] During childhood, patients develop insatiable appetite, hyperphagia and obesity. Mild learning impairment and behavioral problems are also noted including self-injury, obsessive-compulsive disorder, anxiety, and temper tantrums.

Families of children with PWS have reported that weight and behavior issues are of greatest concern. In the 1970s, the protein-sparing modified fast became in use for children with Prader-Willi Syndrome; this diet consisted of 1.5 g of meat protein per kilogram of ideal weight per day, supplemented with low-carb, low-fat foods/drinks [5–11]. More recently, there is also the often used “Red Yellow Green” Weight Management system for children with Prader-Willi Syndrome that emphasizes a balanced diet and avoidance of high-calorie, high-fat foods in addition to environmental control measures [12] .

However, with ketogenic and low carbohydrate diets gaining much interest in the mainstream population, and widely used for refractory pediatric epilepsy, anecdotal reports that this diet was helpful among people with Prader-Willi Syndrome in regards to hyperphagia and weight control surfaced. This study used the Modified Atkins Diet (MAD), which is less restrictive than the classic ketogenic diet used in pediatric epilepsy. MAD allows up to 10–15 g of net carbohydrates (total minus fiber) per day for children, and protein and fat is either unrestricted or can be individualized to achieve ketosis [13–15].

In 2016, the Charlie Foundation and the TREND Community enrolled 14 patients with PWS ages 2 through 11, to complete a modified ketogenic diet for 6 months and report labs and family input on behavior and appetite back to the TREND database [16]. Ten patients completed the 6-month duration of the diet and 4 were unable to due to reasons besides ineffectiveness of the diet. Of those that completed the diet, all 10 participants and families noted positive impact on behavioral/cognitive function, and/or hyperphagia and all of these families continued the diet beyond the study’s 6 month period.

This feasibility study is a prospective study that enrolled children with PWS to complete a 4-month trial of the Modified Atkins Diet, and studied weight changes, laboratory markers, tolerability of the diet and subjective behavioral changes.

## Methodology

This study was approved by the Institutional Review Board at Johns Hopkins University School of Medicine. This was a pilot and feasibility study investigating the Modified Atkins Diet for children with Prader-Willi Syndrome. It was funded by the Foundation of Prader-Willi Research, Canada and a NIH T32 Training Grant. Children ages 6–12 years with genetically confirmed PWS were recruited. Recruitment was done through offering study participation to patients of the principal investigator and social media advertising on the Foundation for Prader-Willi Research page. Breakfast and lunch were provided for the day of study visits for the participant. Travel costs were reimbursed for out-of-state participant families to help offset financial burden. Consent from parents and assent from children were obtained. Study visits were conducted at the Johns Hopkins Hospital Pediatric Clinical Research Unit in Baltimore, Maryland, USA. Participants were excluded from the study if they had a history of hyperlipidemia, multiple food allergies, significant GI dysmotility, or hypercalciuria.

Total study duration was approximately 12 months per participant, with 4 months being on the diet and 4 months being off the diet and an initial teaching session. The study design consisted of three study visits, 4 months apart, at the study site for each participant. At each visit, participants met with the study physician, research dietitian and study psychologist. Each visit consisted of updated history, anthropometry, fasting blood work (comprehensive metabolic panel, lipid profile, hemoglobin A1C, insulin level), urine studies (urinalysis, urine calcium, urine creatinine), and psychology questionnaires. Families and participants were also asked to comment subjectively on behavior, skin picking, and hyperphagia by the study psychologist or study physician.

At the first study visit, the research dietitian met with study participants and their caregivers for a 2 h teaching session on how to implement the study diet and track urine ketones; recipes and sample menus were provided. Study participants were instructed to follow a 10–15 g net carbohydrate limit (a calculation of total carbohydrates minus fiber), to take a general pediatric multivitamin with minerals, a vitamin D supplement (600 international units – IU) daily, and a calcium supplement (1000 mg/day for 4–8 years olds and 1300 mg/day for 9–13 years olds). Protein and fat guidance was provided on an individual basis. Hydration was encouraged.

A power calculation and statistical analysis was not done due to small sample size. The statistics reported are descriptive. The goal of this study was feasibility and it was acknowledged that we would not have the numbers needed to gain statistical power of this data.

## Results

Table 1 shows the basic demographic and clinical information for patients enrolled. Seven participants, age 6–12, with genetically confirmed PWS were enrolled in the study. All except one had a BMI greater than the 95th percentile, but with a considerable range in BMI from 16.7 kg/m2 to 61.9 kg/m2 (height data not shown).

**Table 1:** Clinical Characteristics of Study Participants

Participant No.	Age (years)	Sex	Ethnicity	BMI	BMI %	Genetic Basis
**1**	12.6	F	White	23.9	95%	Uniparental Disomy
**2**	8.1	F	White	22.5	97%	Chromosomal Deletion
**3**	8.9	F	Asian	28.9	99%	Chromosomal Deletion
**4**	7.1	F	White	21.5	97%	Uniparental Disomy
**5**	6.0	M	White	16.7	78%	Uniparental Disomy
**6**	7.6	F	Black	30.8	99%	Chromosomal Deletion
**7**	12.6	M	Black	61.9	> 99%	Chromosomal Deletion

**Fig. 1:**
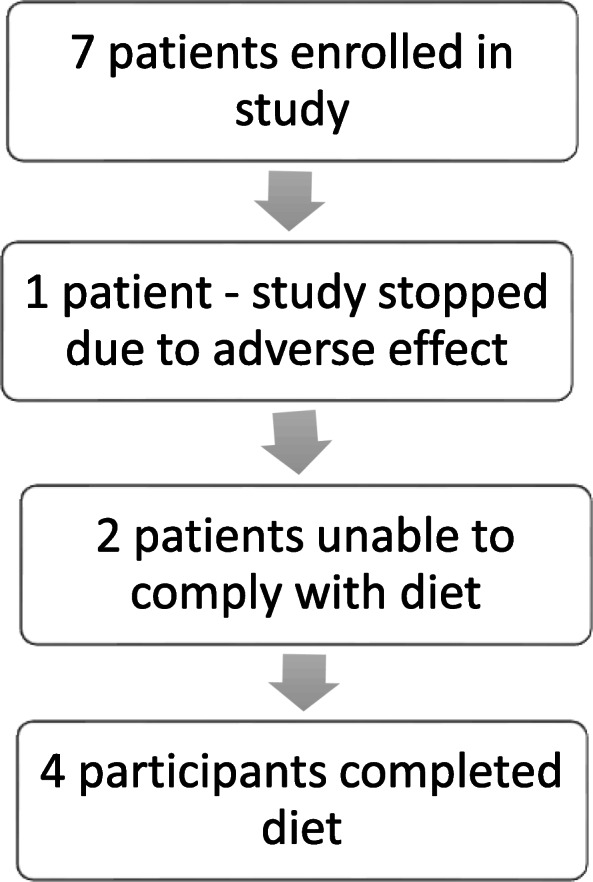
Participants enrolled

Figure 1 explains the flowchart of participants who were enrolled in the study and able to complete the study and exceptions. Two participants were unable to comply with the diet and did not complete the diet. For example, one of these participants attempted the diet while at home but was still having regular school lunches including juice and milk daily. There were also several social stressors for the family at the time. The second participant that was unable to do the diet essentially never started due to family’s difficulty to plan meals and have “allowable foods” available and have all caregivers in the family on board with the diet. One participant had an elevated urine calcium/creatinine ratio prior to the start of the diet so the patient was excluded per protocol. In total, four participants completed the full 4-month diet trial and study.

Participants who did complete the diet reported that it was tolerable and parents saw drastic changes in their child’s attitude toward food. Parents reported that children were not as obsessed about the “next snack or the next meal.” One parent recalled an example: at a birthday party, one participant took “only ¼ of a mini-cupcake to eat at a later time; something that she would never have done before”. They stated that frequency of temper tantrums and moodiness was “much improved”. Parents were hesitant to stop the diet at the end of the 4-month period due to these improvements despite the need for meal planning and initial challenges of the diet. All participants were asked to stop at the 4 month period per the study protocol to assess post-diet changes. After this “off-diet” study period, all four families went back on the Modified Atkins Diet or at least some version of it, i.e. slightly higher allowable total carbohydrates.

### Weight

**Fig. 2:**
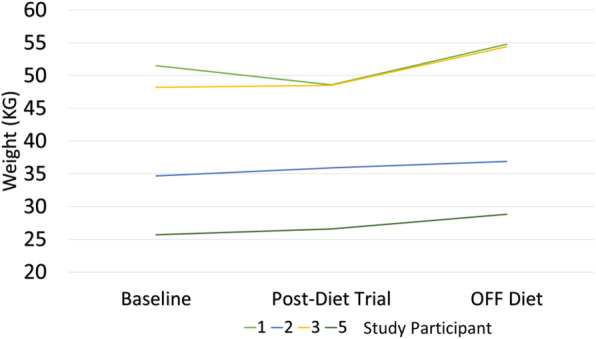
Weight Changes of Participants on and off Diet

Weight loss was not significant on the diet (see Fig. 2). Only one participant had a weight loss of 2.9 kg during the 4-month diet period. The remainder essentially maintained their weight. However, BMI z-scores improved for three out of the four participants, decreasing by average of 0.21 points for each participant. Weight gain was noted in all participants after the diet was stopped, especially the patient who did lose weight, almost in a rebound manner. Height curves were not negatively affected on the diet.

### Laboratory measures

Blood work was checked at baseline, at the end of the diet period and 4 months off the diet. Three of four participants saw an increase of LDL, reflected in total cholesterol level as well. Triglycerides were overall stable; one participant saw a 39 mg/dl decrease in triglycerides.

Regarding markers of metabolic syndrome, Hemoglobin A1C values decreased in 3 out of 4 participants. Homeostasis model assessment of insulin resistance (HOMA-IR) scores were calculated using fasting insulin and glucose levels [17]. Impact on the diet on these scores were variable; one participant saw a profound drop in their HOMA-IR score; in the others, it was either stable or increased. None of the participants had elevated liver enzymes at baseline and these levels remained unchanged with the diet as noted.

Urine calcium/creatinine levels were normal for all participants at the end of the diet period and urinalyses did not demonstrate any crystals or red blood cells for any participant except for the one patient that the study protocol was stopped for due to urinalysis abnormalities (Table 2).

**Table 2:** Laboratory Measures in Pre and Post-Diet Checks

Participant No.	1	2	3	4 (excluded Abnl urine)	5	6 (Dropped)
**Total Cholesterol (mg/dl)**						
Pre-Diet	147	181	197	142	156	116
Post-Diet	136	211	223		296	
Difference	−11	30	26		140	
**LDL (mg/dl)**						
Pre-Diet	73	98	112	80	97	55
Post-Diet	59	135	131		235	
Difference	−14	37	19		138	
**Triglycerides (mg/dl)**						
Pre-Diet	57	29	103	37	81	72
Post-Diet	59	42	64		80	
Difference	2	13	−39		−1	
**Hemoglobin A1C**						
Pre-Diet	5.2	4.8	6	4.9	5.6	5.8
Post-Diet	4.8	4.8	5.7		4.9	
Difference	−0.4	0	−0.3		−0.7	
HOMA-IR Score*						
Pre-Diet	2.8	0.8	12.1	7.42	2.1	18.4
Post-Diet	3.8	1.9	5		2.3	
Difference	1	1.1	−7		0.2	
**ALT (nl < 31 IU)**						
Pre- Diet	14	20	16	28	13	12
On-Diet	14	32	14		26	
Post-Diet	14	17	16		10	
**Urine Ca/Cr Ratio (nl < 0.2)**						
Pre-Diet	0.065	0.12	0.065	0.60	0.06	n/a
On Diet	0.089	0.16	0.031		0.16	
Post-Diet	0 .01	n/a	0.057		n/a	

*HOMA-IR score above 1.9 – early insulin resistance; score above 2.9- significant insulin resistance

Abbreviations: *mg* milligrams, *dl* deciliter, *IU* international units

## Discussion

This is the first prospective clinical study on a low-carbohydrate diet for children with Prader-Willi Syndrome. Compliance to the diet was difficult for two out of seven of our participants, but the remainder found the MAD very feasible. Families reported several challenges to implementing a high-fat/low-carbohydrate diet including necessary meal planning, accommodations at school or needing to pack lunch, garnering acceptance and support from others caring for the child and others in the home (ex. siblings), and the increased financial cost of a high-fat/low-carbohydrate diet.

All four participants that completed the diet had weight stabilization or weight loss. In the off-diet period, weight gain velocity was noted to be increased. Thus, weight stabilization in this typically hyperphagic population can be seen as a positive outcome.

Three out of four of the participants who completed the diet had a significant elevation in their total cholesterol and LDL. This was an expected finding based on larger studies of patients on the modified atkins diet. Cervenka et al., found that among adult patients with epilepsy on the MAD, total cholesterol and LDL increased in the first 3 months and then normalized after 1 year on the diet [18]. A recent study on children with epilepsy on the ketogenic diet demonstrated that although LDL and triglycerides increase on the diet, carotid intima-media thickness and other markers of elasticity of the carotid artery and aorta does not worsen on a high-fat, low-carbohydrate diet [19, 20]. Further long-term studies are needed to establish cardiovascular safety of this diet due to potential benefits for a variety of conditions.

Two out of the seven participants in this small sample had an adverse effect: hypercalciuria and significant hyperlipidemia, respectively. These adverse effects had no clinical effects and resolved off the diet. However, this underlines that patients undertaking this diet for its possible beneficial effects should be followed by a medical team. There was no worsening constipation reported among study participants. No other adverse effects were seen during the diet period.

Limitations of the study include small sample size. Studies looking at diet are inherently difficult due to lack of precise control of actual dietary intake. We asked parents to report participants’ weekly urine ketone checks but to improve accuracy of compliance, we could ask parents to send photos of the urine stick, for example.

## Conclusions

Compliance to restrictive diets can be challenging for children and more so for children with PWS who may be sneaking in food. Additionally, there is likely an aspect of the Hawthorne effect: parents and children both know they are on a diet that is claimed to improve hyperphagia, satiety, behavior and help with weight loss so may report those effects greater than may actually exist. Also, families who had already implemented strict dietary measures to manage their child’s Prader-Willi Syndrome were more likely to join the study and comply with the diet, an inherent selection bias. As more patients with Prader-Willi Syndrome implement this diet, this small pilot study demonstrates justification for larger multi-center studies.

## Data Availability

Supporting data is available for review upon request.
